# Altered Development of NKT Cells, γδ T Cells, CD8 T Cells and NK Cells in a PLZF Deficient Patient

**DOI:** 10.1371/journal.pone.0024441

**Published:** 2011-09-06

**Authors:** Maggie Eidson, Justin Wahlstrom, Aimee M. Beaulieu, Bushra Zaidi, Steven E. Carsons, Peggy K. Crow, Jianda Yuan, Jedd D. Wolchok, Bernhard Horsthemke, Dagmar Wieczorek, Derek B. Sant'Angelo

**Affiliations:** 1 Department of Pediatrics, Memorial Sloan-Kettering Cancer Center, New York, New York, United States of America; 2 Immunology Program, Sloan-Kettering Institute, Memorial Sloan-Kettering Cancer Center, New York, New York, United States of America; 3 Ludwig Center for Cancer Immunotherapy, Immunology Program, Memorial Sloan-Kettering Cancer Center, New York, New York, United States of America; 4 Division of Rheumatology, Allergy and Immunology, Department of Medicine, Winthrop-University Hospital, Mineola, New York, United States of America; 5 Rheumatology Division, Mary Kirkland Center for Lupus Research, Hospital for Special Surgery, New York, New York, United States of America; 6 Weill Graduate School of Medical Sciences of Cornell University, New York, New York, United States of America; 7 Department of Medicine, Memorial Sloan-Kettering Cancer Center, New York, New York, United States of America; 8 Institut fuer Humangenetik, Universitaetsklinikum Essen, Essen, Germany; 9 Gerstner Graduate School of Biomedical Sciences, Memorial Sloan-Kettering Cancer Center, New York, New York, United States of America; University of Hawaii, United States of America

## Abstract

In mice, the transcription factor, PLZF, controls the development of effector functions in invariant NKT cells and a subset of NKT cell-like, γδ T cells. Here, we show that in human lymphocytes, in addition to invariant NKT cells, PLZF was also expressed in a large percentage of CD8+ and CD4+ T cells. Furthermore, PLZF was also found to be expressed in all γδ T cells and in all NK cells. Importantly, we show that in a donor lacking functional PLZF, all of these various lymphocyte populations were altered. Therefore, in contrast to mice, PLZF appears to control the development and/or function of a wide variety of human lymphocytes that represent more than 10% of the total PBMCs. Interestingly, the PLZF-expressing CD8+ T cell population was found to be expanded in the peripheral blood of patients with metastatic melanoma but was greatly diminished in patients with autoimmune disease.

## Introduction

The promyelocytic leukemia zinc finger (PLZF, *ZBTB16*) transcriptional regulator is a member of the BTB/POZ-ZF (Broad complex, tramtrack, bric-à-brac or poxvirus and zinc finger-zinc finger; BTB-ZF) family of proteins that have a wide variety of biological activities [1]. Over the last few years, it has become apparent that BTB-ZF proteins are critical regulators of immune system development and function. For example, Bcl6 has been shown to be necessary for both the B cell germinal center reaction [2], [3] as well as for the development of follicular helper T cells [4], [5]. ThPok has been shown to be necessary and sufficient for CD4 T cell development [6] and the B cell versus T cell commitment step is controlled by LRF (leukemia/lymphoma related factor) [7]. Mazr influences CD8 T cell development, in part by regulating the expression of ThPok [8].

PLZF was first identified due to a t(11;17)(q23;q21) translocation that fused PLZF with RARα(retinoic acid receptor alpha) in some patients with acute promyelocytic leukemia (APL) [9]. The fusion protein that results from this translocation was subsequently shown to be an important determining factor in the development APL and its response to therapy with retinoic acid [10]. The transcription factor has since been implicated in a wide variety of developmental processes, including axial skeletal patterning [11], CNS development [12], male spermatogenesis [13], [14], and immune function [15].

Recently it was shown that PLZF is highly expressed in mouse and human invariant natural killer T (iNKT) cells [16], [17]. NKT cells represent a subset of CD3^+^ lymphocytes that serve as a bridge between the innate and adaptive immune systems. Invariant NKT cells represent a subset that expresses a CD1d-restricted Vα24Jα18 TCR in humans [18], [19]. Both variant and invariant subsets are capable of rapid and copious secretion of a wide variety of Th1 and Th2 cytokines upon initial stimulation, although evidence suggests that the specific roles of each subset may differ [20]. The invariant TCR expressed by iNKT cells recognizes lipid antigens presented in the context of the MHC class I-like molecule CD1d. Invariant NKT cells, which have been extensively studied in mice, are, comparatively, quite rare in humans. For example, in mice 30–40% of T cells in the liver are iNKT cells; in humans only ∼1% of hepatic T cells are iNKT cells [21], [22], [23]. Only 0.008–1.1% of human peripheral blood T cells and only 0.001–0.01% of human thymocytes have been reported to be iNKT cells [24], [25], [26].

Our lab, and others, have shown that in mice, PLZF controls the development of essentially all of the innate-like features of NKT cells [16], [17]. For example, PLZF-deficient NKT cells do not acquire the typical “activated” phenotype characterized by high expression of CD44 and CD69 [16], [17]. PLZF deficient NKT cells also do not constitutively express granzyme B or the mRNA transcript for IL-4 [16] and fail to acquire the capacity to secrete multiple cytokines upon primary stimulation [16], [17]. Furthermore, the frequency of NKT cells is substantially reduced in PLZF-deficient mice and the cells accumulate in the lymph nodes and spleen rather than in the thymus and liver. Overall, the phenotype of PLZF-deficient NKT cells is highly reminiscent of naïve, conventional CD4 T cells [16]. In contrast, ectopic expression of PLZF in conventional T cells results in the acquisition of innate T cell-like characteristics such as an activated phenotype, the rapid secretion of Th1 and Th2 cytokines in response to an initial stimulus and homing to non-lymphoid tissues [17], [27], [28].

Recent studies have shown that PLZF expression is not strictly limited to invariant NKT cells in mice, but can also be found in a specific subset of γδ T cells that express a Vγ1.1Vδ6.3 TCR [29], [30], [31]. This subset of “NKT” γδ T cells functionally resemble invariant NKT cells in that they co-secrete both IFN-γ and IL-4 upon primary activation. Importantly, PLZF has been shown to be required for the innate T cell-like characteristics of NKT γδ T cells [29]. These studies, together with the findings in NKT cells, highlight an essential and non-redundant role for PLZF in the development of innate T cell effector functions.

In addition to directly controlling the function of the cells it is expressed within, PLZF impacts immune function in trans. Of great interest, studies show that the IL-4 produced by these PLZF-expressing cells profoundly alters the CD8 T cell compartment [32], [33]. In mice with an expanded PLZF-expressing T cell compartment, CD8 T cells were found to take on an innate-like phenotype, represented by increased expression of CD44, Eomes and an enhanced capacity to secrete IFN-γ. Such mice also harbor increased numbers of germinal center B cells and high serum levels of IgE, in concordance with their heightened Th2 responses [31]. These data show that innate-like T cells, such as NKT cells, have a broad impact on the immune response.

The role of NKT cells in disease is complex and appears to be dependent on both the NKT cell subtype and the microenvironmental context. In mice, NKT cells have been shown to be important in the suppression of solid tumors as a consequence of interactions with dendritic cells and other lymphocytes [34], [35], [36], [37], [38], [39], [40]. In contrast, the immunomodulatory activity of NKT cells can also influence the immune response against autoantigens. For example, autoimmunity in the Type 1 diabetes-susceptible NOD mouse appears to be exacerbated by a deficiency of invariant NKT cells since it is alleviated by NKT activation [41]. In humans with autoimmune diseases, including systemic lupus erythematosus (SLE) [42], diabetes mellitus, and rheumatoid arthritis, decreases in circulating NKTs have, in some studies, been shown to correlate with the frequency of disease [43].

Spurred by recent reports of a broader expression pattern of, and functional relevance for, PLZF in mouse lymphocytes, we examined the full breadth of PLZF expression in human T cells. Furthermore, analysis of the only known person to harbor a biallelic loss of PLZF enabled us, for the first time, to examine the role of this transcription factor in the development of human T cells. We found that in addition to iNKT cells, nearly all γδ T cells and natural killer (NK cells) expressed PLZF. Furthermore, more than 5% of CD8^+^ T cells and ∼2% of CD4^+^ T cells were found to express the transcription factor. Therefore, in total, more than 10% of human PBLs express PLZF. Finally, to study the importance of PLZF for the development of these various cell types, we obtained peripheral blood samples from the only person known to harbor a biallelic loss of functional PLZF [44]. Overall, our data suggest that differences in PLZF expression represent a significant divergence between the mouse and human immune system.

## Materials and Methods

### Blood Draw

Blood draw protocols and ethics were reviewed and approved by the Internal Review Board's (IRB) at Memorial Sloan-Kettering Cancer Center (New York, NY), the Hospital for Special Surgery (New York, NY), the Winthrop-University Hospital (Mineola, NY) and Universitaetsklinikum Essen (Essen, Germany). Written informed consent forms were obtained from all donors.

### Flow Cytometry

Anti-CD3-Pacific Blue-CD4-PerCPCy5.5, -CD8-AlexaFluor700, CD56-PECy7, -CD161-APC, -γδTCR-PE, -Vα24-Jα18-PE, and –CD4-AlexaFluor488, were obtained from BD, eBioscience or BioLegend. Mags.21F7 (anti-PLZF) was described [16]. Dead cells were excluded by DAPI when possible and doublets were excluded. Cell sorts were done by MSKCC's Flow Cytometry Core Facility. Post-sort analysis confirmed cell purity >95%.

### Cytokine Expression

T cells were plated in X-vivo media with PMA (100 ng/ml) and ionomycin (500 ng/ml) or beads coated with anti-CD3/CD28 (Miltenyi Biotec). Brefeldin A was added for the last five hours of a 6 hour culture. Cells were stained for surface markers, fixed, made permeable and stained with anti-IFNγ AlexaFluor 488 (BD). NK cells were activated with beads coated with anti-NKp46 and anti-CD2 (Miltenyi Biotec). Cytokines were analyzed with cytokine bead arrays (BD).

### Patient Samples

Patients with an established diagnosis of systemic lupus erythematosus (SLE, n = 6), primary Sjögren's syndrome (n = 3), or metastatic melanoma (n = 10) and healthy controls (n = 8) were consented in accordance with local institutional review board guidelines. Lymphocytes were isolated from patients with stage III-IV melanoma by leukapheresis. Lymphocytes from fresh peripheral blood for other samples were isolated by Ficoll extraction.

### Real Time PCR

Sorted cells were suspended in 1 ml Trizol and frozen at -80 degrees. RNA was isolated using Qiagen's RNeasy mini kit. Following reverse transcription, the abundance of PLZF cDNA was quantified by real-time PCR using the MasterMix (2X) HotStart-IT Syber kit (USB). Expression levels were normalized against the housekeeping gene GAPDH, according to the formula: relative PLZF induction  = 2^CT(GAPDH) –^ ^CT(PLZF)^.

### Statistical Methods

Statistical analysis was done either with a two-tailed, unpaired Student's t-test or the Mann-Whitney U-test, which is a more robust for small sample sizes.

## Results

### PLZF expression in human T cells

**Figure 1:**
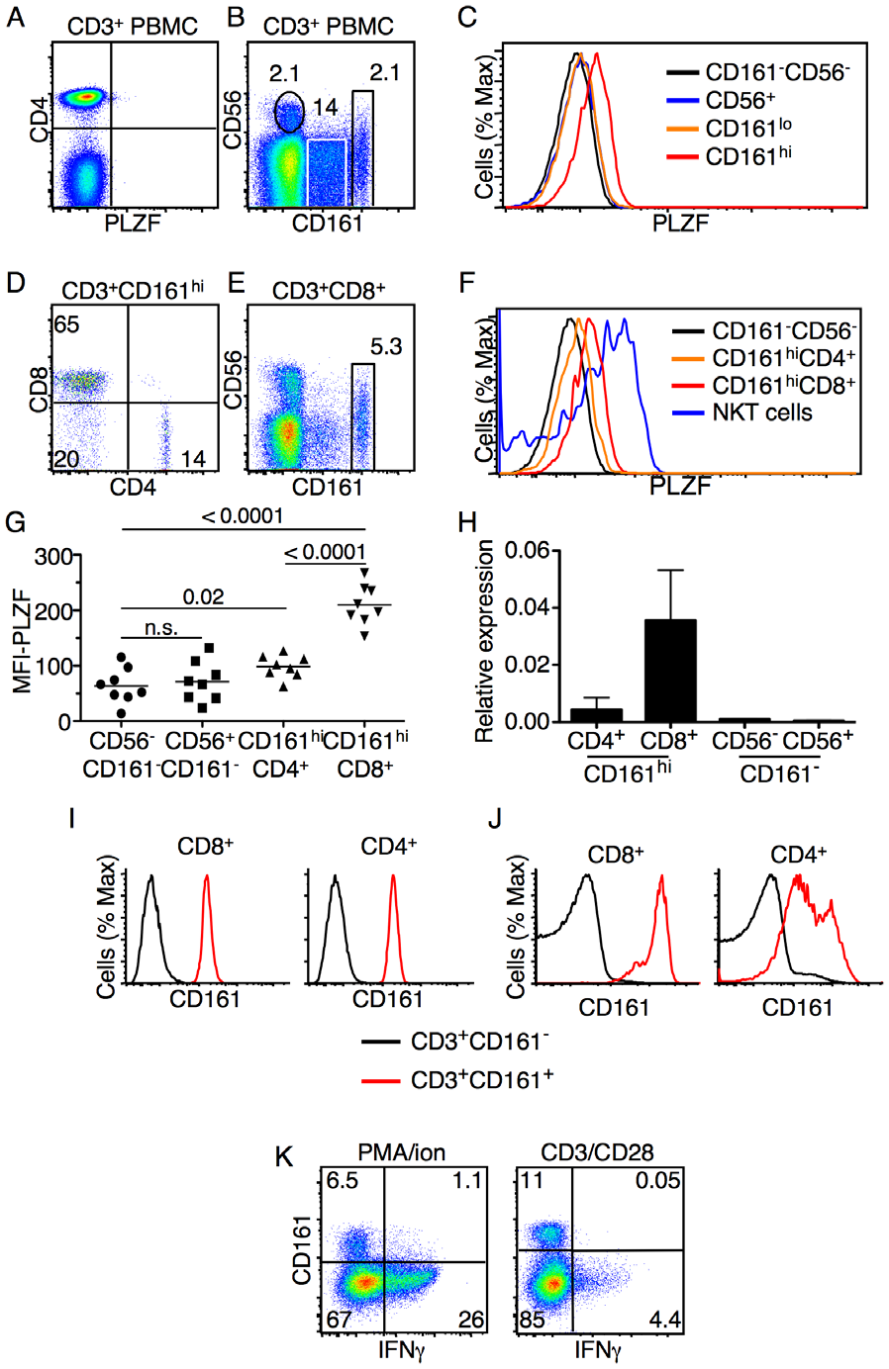
Expression of PLZF in peripheral T cell subsets. (**A**) Intranuclear FACS analysis of PLZF expression in total CD3^+^ T cells; (**B**) CD161 and CD56 expression on CD3^+^, NKT cell^−^, γδ TCR^−^ lymphocytes; (**C**) PLZF expression in the indicated cell populations. Expression is compared to that of PLZF negative CD3^+^CD161^−^CD56^−^ conventional T cells (black line). (**D**) CD4 and CD8 expression on electronically gated CD3^+^CD161^hi^ T cells. (**E**) CD161 expression on electronically gated CD3^+^CD8^+^ T cells. (**F**) PLZF expression in the indicated cell populations. (**G**) Mean fluorescence intensity (MFI) of PLZF expression in indicated cells (N = 8). P values are indicated (n.s.  =  not significant); (**H**) cDNA made from RNA collected from the indicated populations was analyzed by real-time quantitative rtPCR. Units are relative to the expression of GAPDH. Data are the average of two independent experiments with an N = 4. (**I**) Healthy donor lymphocytes were sorted into four T cell subsets: CD161^−^CD4^+^, CD161^−^CD8^+^, CD161^hi^CD4^+^ and CD161^hi^CD8^+^. Representative data for CD161 expression on the four populations analyzed immediately post-sort is shown (black line  =  CD161^−^ cells; red line  =  CD161^hi^ cells). (**J**) CD161 levels of the indicated sorted cells, analyzed three days after activation with anti-CD3/CD28 coated beads, is shown (black line  =  CD161^−^ cells; red line  =  CD161^hi^ cells). (**K**) IFN- γ expression by peripheral blood T cells 6 hours after activation with PMA/Ionomycin or anti-CD3/C28 coated beads. Numbers indicate percentage of cells within the indicated gate. Data in A–G are representative of eight donors, each analyzed at least two times. Data in I and J are representative of two individuals done in two independent experiments. Data shown in K are from two different individuals and were two independent experiments.

PLZF is expressed both in mouse and human invariant NKT cells [16]. Recent studies in mice, however, show that PLZF expression is not limited to this cell type [29], [30], [31]. Therefore, we more carefully examined the expression of PLZF in PBLs from healthy volunteers by FACS. Similar to mice, PLZF was not detected in “conventional” T cells (**Fig. 1A**). The γδ TCR and iNKT cell-negative, CD3^+^ T-cell compartment can be demarcated by the expression of CD161 and CD56. These cells are found at fairly high frequencies, with CD56^+^ T cells on average at 1.7% (+/-2.5%) and CD161^+^ T cells often found at more than 10% of the total T cells (**Fig. 1B**). CD56^+^ T cells, which are often are considered to be non-invariant NKT cells, did not express PLZF (**Fig. 1C**). Similarly, CD161^lo^ cells (white box, **Fig. 1B**) also did not express PLZF. Of particular interest, however, was a distinct population of T cells (3.4% +/−2.5%) marked by high levels of CD161 (**Fig. 1B**) that clearly expressed PLZF (**Fig. 1C**).

The majority of the PLZF-expressing CD161^hi^ T cells were found to be CD8^+^ (**Fig. 1D**). Furthermore, the CD161^hi^CD8^+^ T cells (**Fig. 1E**), expressed higher levels of PLZF as compared to the CD161^hi^CD4^+^ T cells (**Fig. 1F**). PLZF expression was lower than in iNKT cells (**Fig. 1F**). PLZF expression levels were consistent among cells from eight healthy donors (**Fig. 1G**). Expression levels were also confirmed by rtPCR of sorted cell populations (**Fig. 1H**). The CD161^hi^CD8^+^ population represented, on average, ∼6% of the total CD8^+^ T cells (**Figs. 1B, 1E**). PLZF is not expressed in any identified CD8^+^ T cell population in mice [**16**]and unpublished data). Therefore, PLZF expression defines an abundant T cell subgroup in humans that does not exist in mouse peripheral blood.

To determine if the CD161^hi^ subset arose from the CD161^−^ population as a result of activation, CD8^+^ and CD4^+^ T cells that were CD161^−^ (**Fig. 1I**, black lines) or CD161^hi^ (**Fig. 1I**, red lines) were sorted and cultured with beads coated with antibodies against CD3 and CD28. After three days, low levels of CD161 were induced on the CD161^−^ T cells (**Fig. 1J**, black lines). CD161^hi^ CD8^+^ T cells were found to retain high levels of CD161 expression post-activation (**Fig. 1J**, red lines). The CD161^hi^ CD4^+^ T cells, however, downregulated CD161 post-activation (**Fig. 1J**, red lines). Therefore, the CD161hi phenotype is specific to the PLZF-expressing CD8^+^ T cells and expression of high levels of CD161 cannot be induced by activation. Furthermore, PLZF was not induced in non-PLZF expressing T cells by activation (data not shown).

Next we activated PBMCs with either PMA/ionomycin or anti-CD3 and anti-CD28, followed by FACS analysis for IFN- γ production. Surprisingly, unlike PLZF-expressing cells in mice [16], [29], few PLZF-expressing CD161^hi^CD8^+^ T cells produced IFN-γ (**Fig. 1K**). As expected, many PLZF-negative CD161^−^CD8^+^ T cells produced IFN-γ upon stimulation (**Fig. 1K**).

### The frequency of PLZF-expressing CD161^hi^CD8^+^ T cells is altered in patients with autoimmunity or advanced melanoma

**Figure 2:**
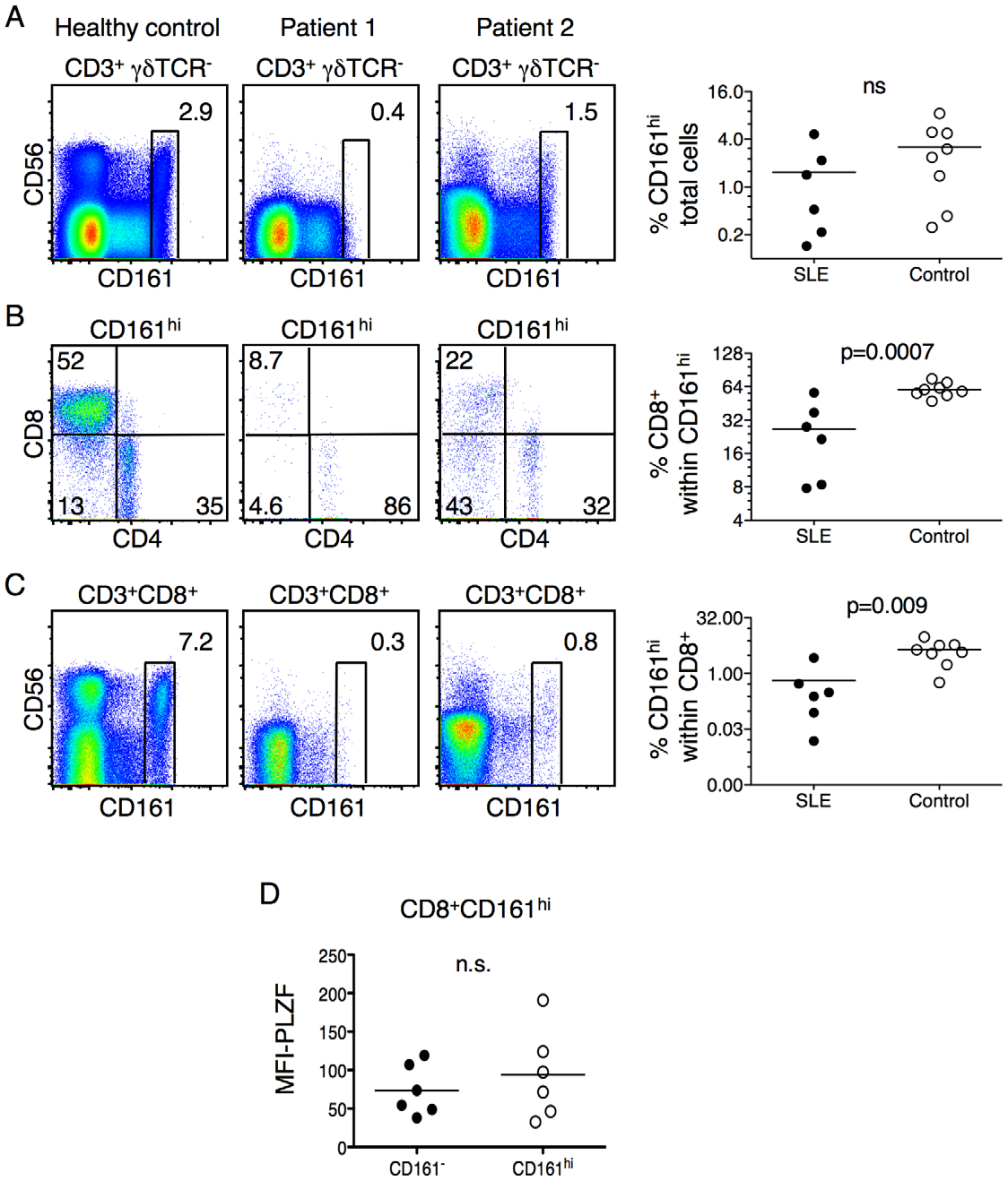
Patients with systemic lupus erythematosus show decreased circulating have altered frequencies of PLZF-expressing T cells. (**A**) The frequency of CD161^hi^ lymphocytes within the CD3^+^ γδ TCR^−^ population. (**B**) The CD4/CD8 profile of the CD161^hi^ T cells as identified in (A). (**C**) The frequency of CD161^hi^ T cells within the CD3^+^CD8^+^ γδ TCR^−^ lymphocyte gate. (**D**) The remaining CD161^hi^CD8^+^ T cells in SLE patients express low levels of PLZF. PBMC's were collected from six patients with SLE and stained as previously described. A representative control and two patient samples are shown for each analysis. Data from the control and patient groups are summarized in scatter plots. P values are shown in figures (n.s.  =  not significant).

To begin to study the role of PLZF-expressing CD161^hi^CD8^+^ T cells in the human immune response, we obtained peripheral blood samples from patients with systemic autoimmune diseases or malignancy. Patient samples were collected based only on availability; no selection criteria were utilized and treatment status was unknown. Monocyte-depleted PBMCs were stained with antibodies against CD3, CD4, CD8, CD161, CD56 and γδ TCR, then analyzed by flow cytometry. Two representative samples acquired from patients diagnosed with systemic lupus erythematosus (SLE) is shown in **Fig. 2A**. CD161^hi^ T cells appeared to be reduced, but not to a level that reached statistical significance, when the SLE samples were compared to healthy donors (**Fig. 2A**). The apparent reduced frequency of CD56^+^ T cells also did not reach statistical significance (data not shown). There was, however, a clear reduction in the percentage of CD8^+^ cells within the CD161^hi^ population in patients with SLE (**Fig. 2B**). Similarly, the frequency of CD161^hi^ cells within the CD3^+^CD8^+^ T cell population was significantly reduced in the SLE samples, as compared to healthy controls (**Fig. 2C**). Again, the apparent reduction of CD3^+^CD8^+^CD56^+^ was not statistically significant (data not shown). A similar trend was seen in a limited survey of patients with primary Sjogren's syndrome (data not shown). Finally, the residual CD161^hi^CD8^+^ T cells in the SLE patients expressed little or no PLZF (**Fig. 2D**).

**Figure 3:**
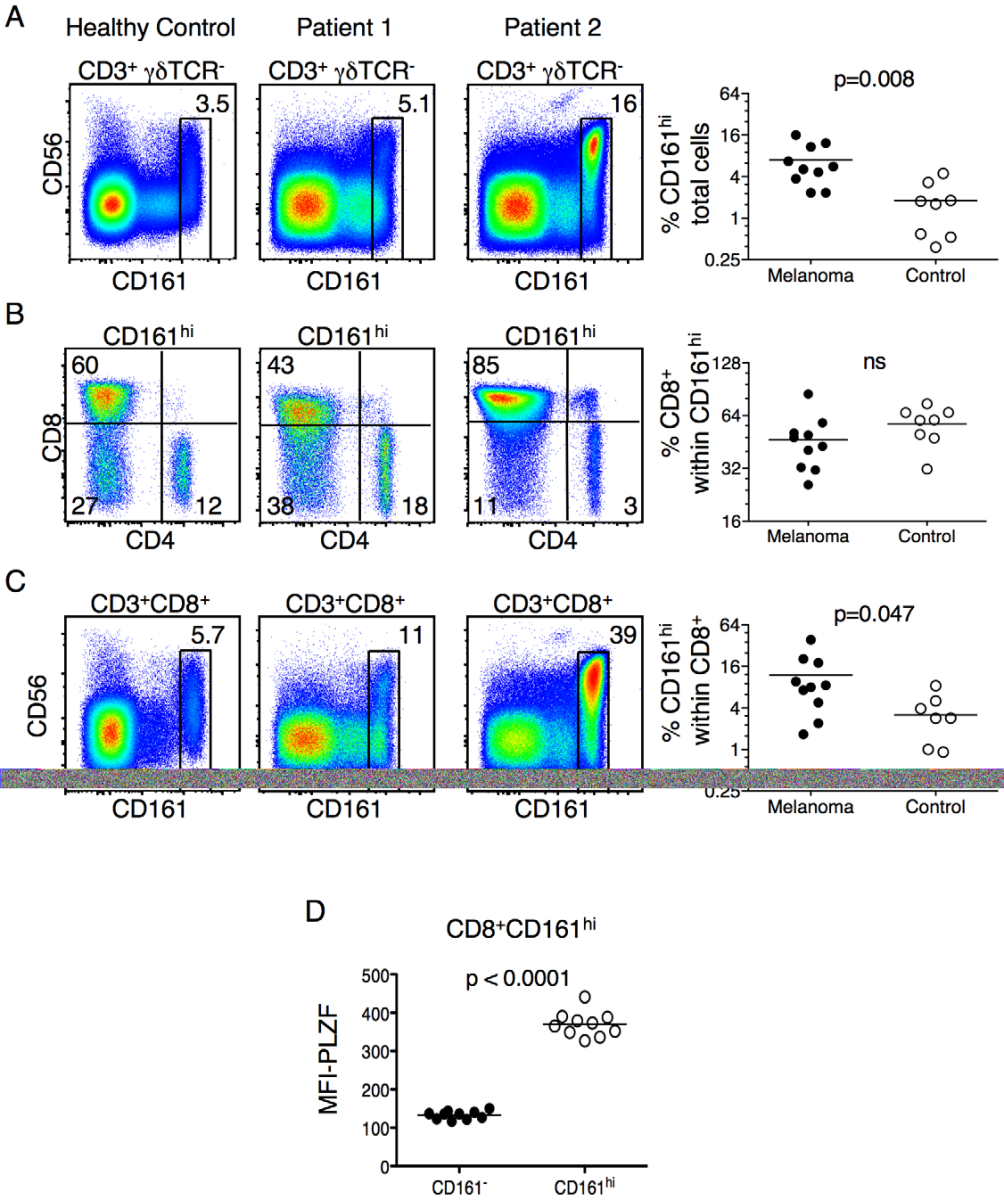
Patients with advanced melanoma have expanded circulating CD8^+^ CD161^hi^ T cell populations. (**A**) The frequency of CD161^hi^ lymphocytes within the CD3^+^ γδ TCR^−^ population. (**B**) The CD4/CD8 profile of the CD161^hi^ T cells as identified in (A). (**C**) The frequency of CD161^hi^ T cells within the CD3^+^CD8^+^ γδ TCR^−^ lymphocyte gate. (**D**) CD161^hi^CD8^+^ T cells from patients with advanced melanoma retain PLZF expression, as compared to conventional CD8 T cells. PBMC's from ten patients with metastatic melanoma were isolated via leukapheresis. The cells were stained and analyzed by FACS as described above. A representative control and two patient samples are shown for each analysis. Data from the control and patient groups are summarized in scatter plots. P values are shown in figures (n.s.  =  not significant).

Next, we evaluated the frequency of CD161^+^ T-cells in blood samples from ten patients with advanced melanoma. In contrast to our findings in SLE and Sjogren's patients, we observed a substantial increase in the frequency of CD161^hi^ T cells in the melanoma cohort as compared to healthy controls (**Fig. 3A**). Different than the patients with autoimmunity, there was no change in the frequency of the CD8 to CD4 ratio within the CD161^hi^ T cells (**Fig. 3B**). Among the CD8^+^ T cells, however, there was a substantial increase in the percentage of CD161^hi^ T cells (**Fig. 3C**). Also different than the SLE samples, the CD161^hi^CD8^+^ T cells in the patient samples were found to express PLZF at levels similar to healthy donors (**Fig. 3D**). These data, along with published studies [45], show that the PLZF-expressing PBL T cell population changes in patients with disease.

### T cell subset deficiencies as a consequence of a biallelic loss of functional PLZF

**Figure 4:**
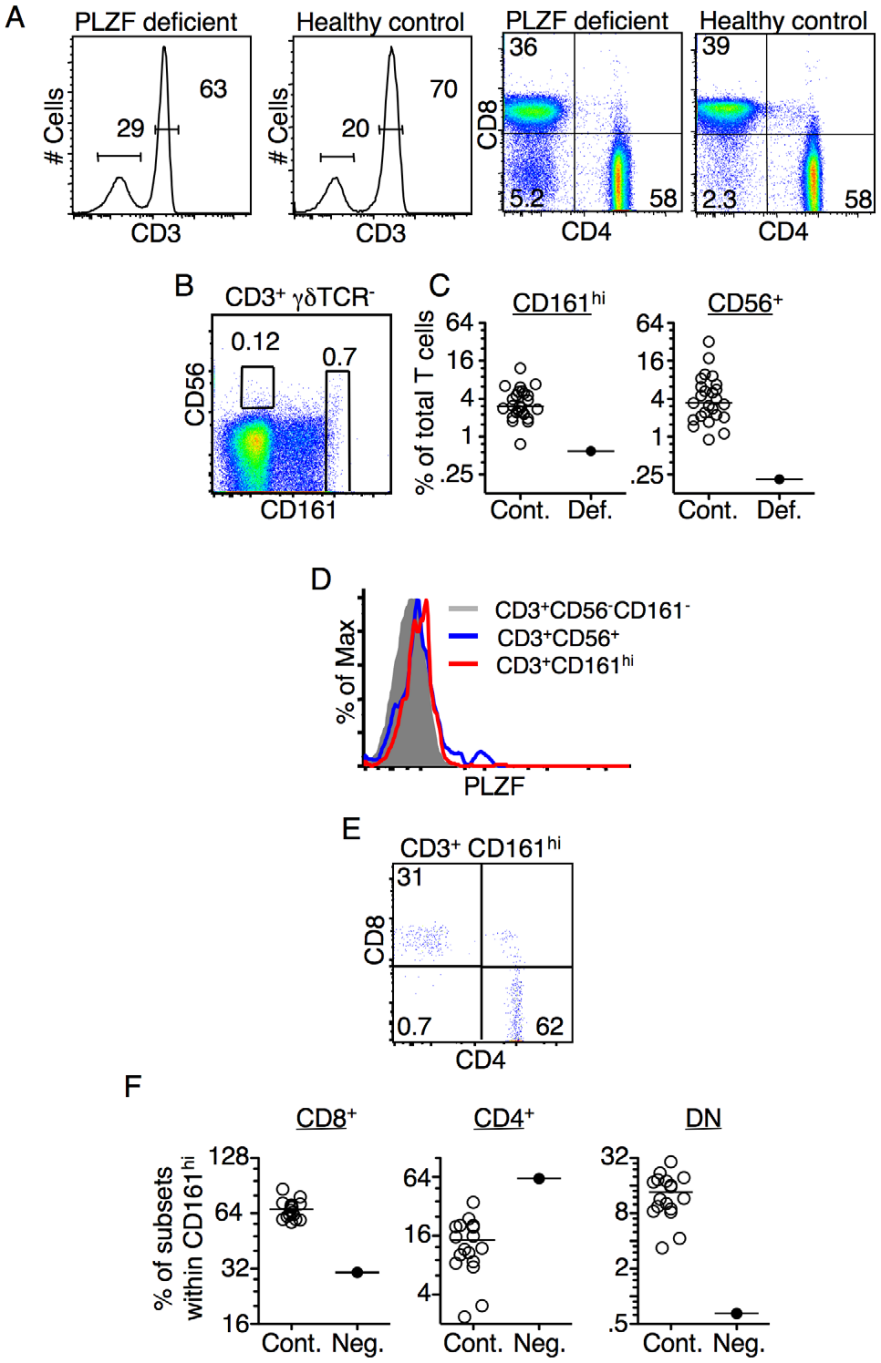
PBMCs from a person with a biallelic loss of functional PLZF were analyzed by FACS. (**A**) The frequency of CD3^+^ T cells and the CD4/CD8 of the CD3^+^ T cells was not altered as compared to healthy donor samples. Results shown are representative of three experiments. (**B**) Dot plot shows the frequency of CD161^hi^ and CD56^+^ expression on CD3^+^ T cell from the PLZF-deficient donor. γδ T cells were excluded from the analysis. (**C**) Scatter plot shows the frequency of the CD161^hi^ and CD56^+^ T cell subsets from the PLZF-deficient donor in comparison to healthy controls (Cont. =  healthy control; Def.  =  PLZF-deficient). (**D**) Histogram shows PLZF expression in CD3^+^ CD161^hi^ and CD56^+^ T cells from the PLZF deficient donor in comparison to the donor's conventional T cells (CD56^−^CD161^−^). (**E**) CD8 versus CD4 expression on NKT cell^−^, γδ T cell^−^, CD161^hi^ T cells from the PLZF-deficient donor. (**F**) Scatter plot shows CD8, CD4 and DN distribution of CD161^hi^ T cells from the PLZF-deficient donor as compared to healthy controls. Numbers within FACS plot represent the percentage of events in the indicated gate. Results were consistent in three samples that were obtained from the PLZF deficient donor.

Recently, the first and, to our knowledge, only person with a biallelic loss of functional PLZF was identified [44]. The maternal allele of PLZF was found to have a missense mutation that resulted in a methionine to valine substitution within a zinc finger domain. This mutation is paired with the paternal chromosome harboring a *de novo* deletion of ∼8 Mbp, resulting in the loss of the gene encoding PLZF. Peripheral blood from this person was obtained and analyzed by FACS. In the absence of PLZF, the total percentage of CD3^+^ T cells among PBLs and the percentage of CD4^+^ and CD8^+^ T cells were similar to healthy controls (**Fig. 4A**). The frequency of the CD56^+^ and the CD161^hi^ T cell populations, however, were reduced as compared to the mean frequency found in healthy controls (**Fig. 4B,C**) and the few CD56^+^ and CD161^hi^ cells found did not express PLZF (**Fig. 4D**). The few CD161^hi^ cells observed in this donor were strongly skewed towards CD4 rather than CD8 expression, which contrasted with the pattern observed in healthy controls (**Fig. 4E,F**). Additionally, there was a decrease in frequency of CD161^hi^ DN T cells, as compared to controls (**Fig. 4E,F**).

### Human iNKT cells require PLZF for normal development

**Figure 5:**
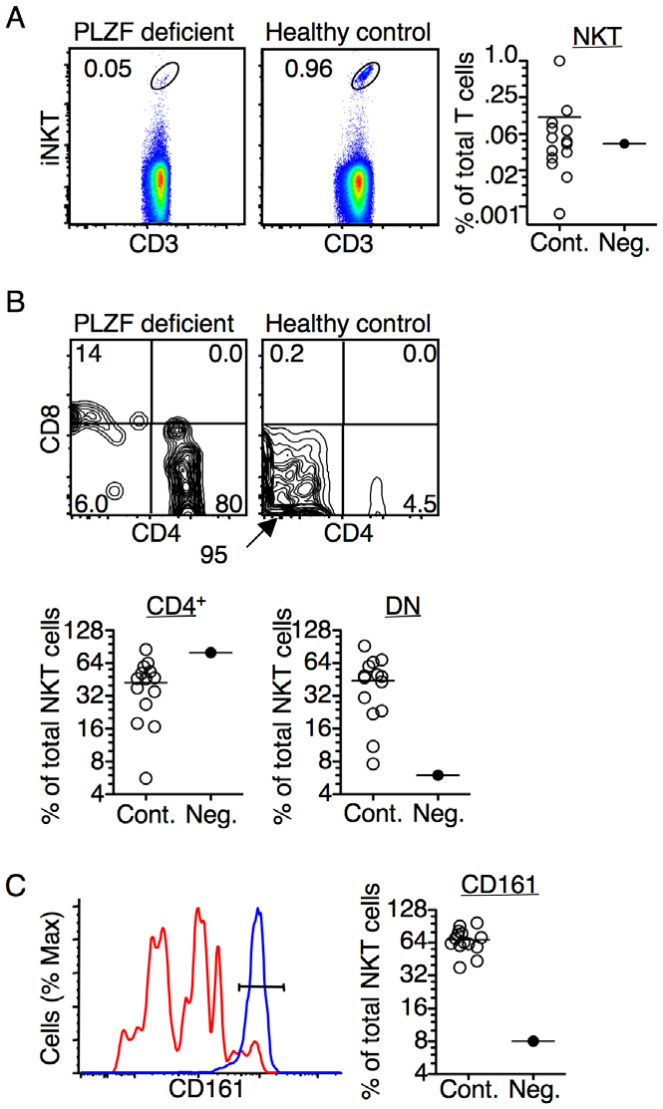
Invariant NKT cell development is altered in the absence of PLZF. (**A**) Invariant NKT cells from the PLZF-deficient and a healthy control donor identified with antibodies against the Vα24Jα18 iTCR antibody (6B11) and CD3. The scatter plot shows the frequency of iNKT cells in the PLZF-deficient as compared to healthy donors. The healthy control sample shown in the dot plot was acquired in the same experiment as the PLZF deficient sample. Therefore, this sample is shown although the frequency of iNKT cells is higher than the median for all samples. (**B**) Dot plots show the expression of CD4 and CD8 on iNKT cells identified in (A). The scatter plots show the frequency of CD4^+^ and CD4^−^CD8^−^ double negative (DN) iNKT cells from the PLZF-deficient donor as compared to healthy controls. (**C**) Histogram shows a comparison of CD161 expression on iNKT cells from the PLZF-deficient donor to healthy controls. iNKT cells were identified as shown in (A). The scatter plot compares the frequency of CD161^hi^ iNKT cells from the PLZF-deficient donor as compared to healthy controls. High expression of CD161 was defined by the black bar shown in the histogram. Cont. =  health control; Neg.  =  PLZF deficient.

In mice, iNKT cells develop in the absence of PLZF, but they do not acquire innate T cell characteristics [16], [17]. For example, in mice, PLZF-deficient iNKT cells accumulate in the lymph nodes rather than in the liver, do not express high levels of CD44 and CD69 and, also, fail to make cytokines upon primary activation. PLZF-deficient mouse iNKT cells are highly skewed towards CD4 and express low levels of NK1.1 [16]. iNKT cells are also present in the peripheral blood of PLZF-deficient mice, but at a reduced frequency as compared to wild type mice (data not shown). The frequency of human iNKT cells in the PLZF-deficient donor was only slightly reduced as compared to the mean frequency found in healthy controls (**Fig. 5A**). However, the phenotype of the PLZF-deficient iNKT cells clearly resembled that of iNKT cells from PLZF-deficient mice. In particular, PLZF-deficient human iNKT cells were strongly skewed towards expressing CD4 at the expense of the DN population (**Fig. 5B**). Furthermore, the PLZF deficient iNKT cells were largely CD161 (NK1.1) low (**Fig. 5C**). These data suggest that, similar to the findings in mice, PLZF impacts the development of iNKT cells in humans.

### Human γδ T cells express PLZF

**Figure 6:**
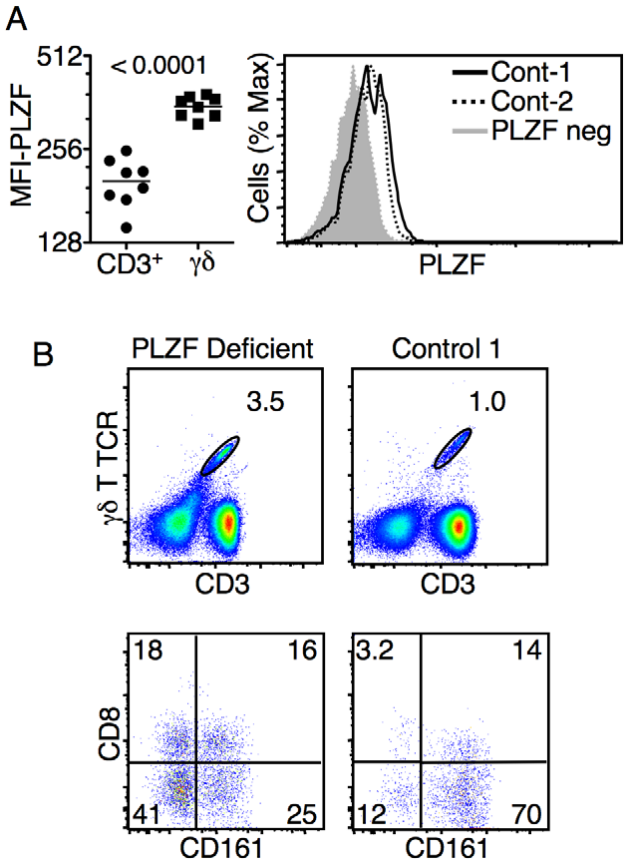
Human γδ T cells express PLZF. (**A**) The MFI of PLZF expression in γδ T cells as compared to CD3^+^CD56^−^CD161^−^ T cells (P value is shown). Histogram shows PLZF expression in γδ T cells collected from two healthy donors in comparison to γδ T cells from the PLZF-deficient donor. (**B**) The frequency of γδ T cells from a healthy control and the PLZF deficient donor is shown in the panels at the top. Although the frequency of γδ T cell in PLZF deficient sample appears higher than the controls, the percentage is actually consistent with a broader analysis (data not shown) and with published reports. The bottom panels show CD161 and CD8 expression on γδ T cells from the healthy control and the PLZF deficient donor. γδ T cells were identified as shown in the top panels. Data from PLZF deficient donor were consistent from three independent blood samples. Numbers indicate the frequency of cells within each panel.

The reduction in the frequency of CD161^hi^ DN T cells in the absence of PLZF (**Fig. 4E,F**) prompted us to more carefully examine this population of T cells. A large percentage of the CD161^hi^ DN T cells proved to be γδ T cells (data not shown). We, and others, recently reported the existence of a minor subset of NKT cell-like γδ T cells in mice that express high levels of PLZF [30], [31]. Unlike the subset-restricted pattern of PLZF expression in mouse γδ T cells, all human peripheral blood γδ T cells were found to express PLZF (**Fig. 6A**). The expression of PLZF was confirmed by comparing γδ T cells from healthy donors to γδ T cells from the PLZF-deficient donor (**Fig. 6A**). γδ T cells were found to develop in the absence of PLZF and were present at a frequency similar to that published by others (∼4%) [46] and to a larger panel of samples we analyzed (**Fig. 6B** **and data not shown**). The data suggests that the frequency of CD161^hi^ γδ T cells was reduced in the absence of PLZF, while the frequency of CD8^+^ γδ T cells was increased (**Fig. 6B**), however, γδ T cell phenotypes were found to be highly variable among healthy donors.

### Human NK cells express PLZF

**Figure 7:**
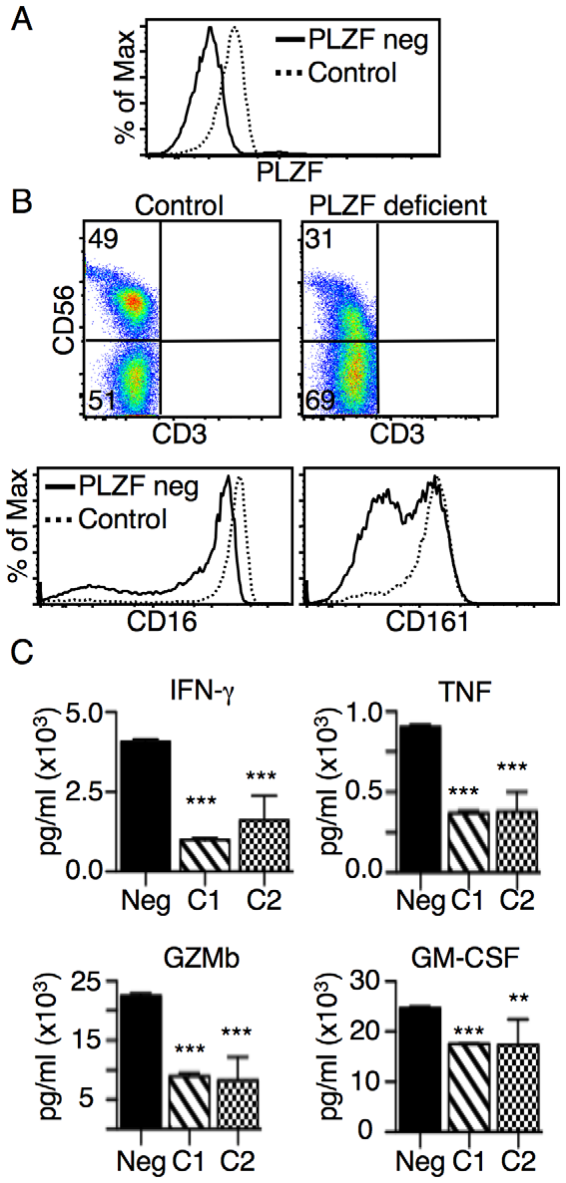
PBMCs from a healthy control and the PLZF-deficient donor were analyzed by FACS. (**A**) Histogram shows PLZF levels in permeabilized CD3^−^CD56^+^ NK cells. (**B**) CD56 levels on CD3^−^ PBMCs from a healthy control or the PLZF-deficient donor. CD16 and CD161 levels on CD3^−^CD56^+^ NK cells (solid line  =  PLZF-deficient donor; dotted line  =  healthy control). (**C**) CD3^−^CD56^+^ NK cells were sorted and then activated with beads coated with anti-NKp46 and anti-CD2. Supernatants were collected after 72 hours and analyzed by cytokine bead array. Control-1 was obtained from a healthy donor the day of the sort; control-2 was obtained from a healthy donor and the sample was shipped with the PLZF-deficient sample. FACS analysis was consistent for eight healthy controls. Samples from the PLZF-deficient donor were collected three times and were analyzed in three independent FACS experiments. Cells for the cytokine release experiment were collected from one sample. Sorted cells were split into multiple wells (between 3 and 8 wells) and treated as independent samples for statistical purposes. (** P value  = 0.0080; *** P value <0.0001).

Less than 5% of mouse NK cells constitutively express PLZF [16] and PLZF is not induced in mouse NK cells by activation (data not shown). Furthermore, mouse NK cells develop and appear to function normally in PLZF-deficient mice ([16] and data not shown). Therefore it was very surprising to find that nearly all human NK cells expressed the transcription factor. The expression of PLZF was made clear by comparing samples from healthy donors to the PLZF-deficient donor (**Fig. 7A**)

PLZF-deficient NK cells were found to express lower levels of CD56, CD16 and CD161 (**Fig. 7B**). To assess the functional relevance of PLZF in human NK cells, NK cells from healthy controls and the PLZF-deficient donor were activated with beads coated with antibodies against CD335 (NKp46) and CD2. Supernatants were collected after three days for cytokine analysis. There was a clear increase in the amount of secreted IFN- γ, TNF and Granzyme B in the PLZF-deficient sample as compared to the control samples (**Fig. 7C**). GM-CSF was also found to be increased, but to a lesser degree. Therefore, PLZF expression appears to subdue cytokine secretion by human NK cells.

## Discussion

We show that in people, CD8^+^ and CD4^+^ CD161^hi^ T cells, all γδ T cells, NKT cells and all NK cells express PLZF. Collectively, these populations represent more than 10% of the total human peripheral blood lymphocytes. In contrast, PLZF expression in mice is limited to NKT cells and a minor subset of γδ T cells, which combined, account for ∼1% of the total lymphocytes. Furthermore, few, if any, mouse NK cells express PLZF. Therefore, PLZF arguably plays a far more significant role in the human immune system than in that of the mouse.

The frequency, phenotype and/or function of all of the PLZF-expressing cell types were altered in some way in samples obtained from a person with a biallelic loss of functional PLZF. For example, the phenotype of CD1d-restricted NKT cells was altered and the frequency of CD161^hi^CD8^+^ T cells was reduced, suggesting that these T cells are dependent upon PLZF for development. γδ T cells were present in the PLZF-deficient donor, but appeared to have altered CD161 and CD8 expression. In the absence of PLZF, NK cells expressed lower levels of CD56 and the activating receptors CD16 and CD161. Surprisingly, we found that PLZF-deficient NK cells produced substantially greater amounts of several different cytokines. No obvious alterations in the PLZF-expressing cell types were detected in a limited analysis of the donor's mother, who carries a point mutation in one copy ZBTB16 (data not shown). As mentioned above, the donor's father carries two non-mutated copies of the PLZF gene and, therefore, blood samples were not obtained. Finally, the donor has not shown overt signs of autoimmunity nor increased susceptibility to infectious disease. Indeed, although the donor is EBV positive, he does not display signs of active disease.

Rather surprisingly, we found that PLZF was not expressed in CD3^+^CD56^+^ T cells. Nevertheless, the frequency of CD3^+^CD56^+^ T cells was reduced in the PLZF-deficient donor, implicating PLZF in the development of these cells. It is possible that expression of PLZF in CD3^+^CD56^+^ T cells may occur during T cell development, when it directs the acquisition of an innate T-cell phenotype in this population, but is then downregulated prior to the migration of these cells into the periphery, similar to what was found for embryonic CD4 T cells [47]. Alternatively, the reduced frequency of CD56^+^ T cells may be due to cell extrinsic factors, such as altered cytokine expression by other PLZF-expressing cells.

Recently published data suggests that a large percentage of CD161^hi^ CD8^+^ and CD161^hi^ double negative T cells express an invariant Vα7.2 TCRα chain [48]. T cells expressing this invariant TCR belong to a subset known as mucosal-associated invariant T cells (MAIT cells). The finding that human, but not mouse, MAIT cells express PLZF suggests a functional divergence in this T cell subset.

To begin to understand the role of PLZF-expressing CD161^hi^CD8^+^ T cell lineage in the human immune system, we analyzed the frequency of this population in blood obtained from patients with either autoimmunity or cancer. Patients with SLE were found to have a highly significant decrease in the percentage of CD8^+^ T cells within the CD161^hi^ subpopulation. Similar results were obtained from a small cohort of patients with primary Sjögren's syndrome. Interestingly, patients with advanced melanoma were found to have a significant increase in the frequency of CD161^hi^ T cells. In one extreme example, ∼16% of the total T cells were CD161^hi^ and, in particular, nearly 40% of the patient's CD8 T cells were CD161^hi^. While these data have not been correlated with clinical outcomes, it is clear that the PLZF-expressing population is dynamic and that it changes with different types of immunological stress.

The patient data are consistent with a previous report showing that CD161^hi^ CD8^+^ T cells are reduced in patients with a variety of rheumatic diseases including SLE, MCTD (mixed connective-tissue disease), SSc (systemic sclerosis) and PM/DM (polymyositis/dermatomyositis) [45]. Our data are also consistent with a report showing that CD161^hi^CD8^+^ T cells do not produce IFN-γ, TNF-α or IL-2 following TCR activation [49]. A more recent paper suggests that CD161^hi^CD8^+^ T cells contain an IL-17 producing subset as well as IL-22 producing cells [50]. Interestingly, it was found that the IL-17 producing cells were enriched in the livers of patients during chronic hepatitis. The PLZF-expressing CD161^hi^CD8^+^ T cells also express high levels of the IL-18 receptor α chain (data not shown). Therefore it is possible that the cells we study here are the same or similar to those described as having the capacity to efflux and, therefore, survive treatments involving chemotherapy drugs [51]. It was suggested that the CD161^hi^ T cells are actually precursors of CD161^lo^ cells and, thus, might represent self-renewing stem cell-like memory CD8 T cells [51]. It will be interesting to determine if these proposed functions are dependent on PLZF.

Overall, we have found that PLZF is expressed in a greater variety of lymphocytes and in a higher percentage of lymphocytes in humans as compared to mice. The function of PLZF-expressing cells appears to be diverse. For example, CD161^hi^CD8^+^ T cells clearly do not make abundant levels of cytokines following activation. Furthermore, PLZF expression in human NK cells appears to limit cytokine production. Therefore, in sharp contrast to other PLZF-expressing lymphocytes, PLZF expression in human CD8^+^ T cells and NK cells appears to inhibit cytokine expression.

## References

[1] Kelly KF, Daniel JM (2006). POZ for effect--POZ-ZF transcription factors in cancer and development.. Trends Cell Biol.

[2] Dent AL, Shaffer AL, Yu X, Allman D, Staudt LM (1997). Control of inflammation, cytokine expression, and germinal center formation by BCL-6.. Science.

[3] Fukuda T, Yoshida T, Okada S, Hatano M, Miki T (1997). Disruption of the Bcl6 gene results in an impaired germinal center formation.. J Exp Med.

[4] Nurieva RI, Chung Y, Martinez GJ, Yang XO, Tanaka S (2009). Bcl6 mediates the development of T follicular helper cells.. Science.

[5] Yu D, Rao S, Tsai LM, Lee SK, He Y (2009). The transcriptional repressor Bcl-6 directs T follicular helper cell lineage commitment.. Immunity.

[6] He X, Park K, Kappes DJ (2010). The role of ThPOK in control of CD4/CD8 lineage commitment.. Annu Rev Immunol.

[7] Maeda T, Merghoub T, Hobbs RM, Dong L, Maeda M (2007). Regulation of B versus T lymphoid lineage fate decision by the proto-oncogene LRF.. Science.

[8] Sakaguchi S, Hombauer M, Bilic I, Naoe Y, Schebesta A (2010). The zinc-finger protein MAZR is part of the transcription factor network that controls the CD4 versus CD8 lineage fate of double-positive thymocytes.. Nat Immunol.

[9] Scaglioni PP, Pandolfi PP (2007). The theory of APL revisited.. Curr Top Microbiol Immunol.

[10] He LZ, Guidez F, Tribioli C, Peruzzi D, Ruthardt M (1998). Distinct interactions of PML-RARalpha and PLZF-RARalpha with co-repressors determine differential responses to RA in APL.. Nat Genet.

[11] Barna M, Hawe N, Niswander L, Pandolfi PP (2000). Plzf regulates limb and axial skeletal patterning.. Nat Genet.

[12] Avantaggiato V, Pandolfi PP, Ruthardt M, Hawe N, Acampora D (1995). Developmental analysis of murine Promyelocyte Leukemia Zinc Finger (PLZF) gene expression: implications for the neuromeric model of the forebrain organization.. J Neurosci.

[13] Buaas FW, Kirsh AL, Sharma M, McLean DJ, Morris JL (2004). Plzf is required in adult male germ cells for stem cell self-renewal.. Nat Genet.

[14] Costoya JA, Hobbs RM, Barna M, Cattoretti G, Manova K (2004). Essential role of Plzf in maintenance of spermatogonial stem cells.. Nat Genet.

[15] Doulatov S, Notta F, Rice KL, Howell L, Zelent A (2009). PLZF is a regulator of homeostatic and cytokine-induced myeloid development.. Genes Dev.

[16] Kovalovsky D, Uche OU, Eladad S, Hobbs RM, Yi W (2008). The BTB-zinc finger transcriptional regulator PLZF controls the development of invariant natural killer T cell effector functions.. Nat Immunol.

[17] Savage AK, Constantinides MG, Han J, Picard D, Martin E (2008). The transcription factor PLZF directs the effector program of the NKT cell lineage.. Immunity.

[18] Bendelac A, Savage PB, Teyton L (2007). The biology of NKT cells.. Annu Rev Immunol.

[19] Kronenberg M, Gapin L (2002). The unconventional lifestyle of NKT cells.. Nat Rev Immunol.

[20] Berzofsky JA, Terabe M (2009). The contrasting roles of NKT cells in tumor immunity.. Curr Mol Med.

[21] Benlagha K, Weiss A, Beavis A, Teyton L, Bendelac A (2000). In vivo identification of glycolipid antigen-specific T cells using fluorescent CD1d tetramers.. J Exp Med.

[22] Matsuda JL, Naidenko OV, Gapin L, Nakayama T, Taniguchi M (2000). Tracking the response of natural killer T cells to a glycolipid antigen using CD1d tetramers.. J Exp Med.

[23] Hammond KJ, Pellicci DG, Poulton LD, Naidenko OV, Scalzo AA (2001). CD1d-restricted NKT cells: an interstrain comparison.. J Immunol.

[24] Marsh RA, Villanueva J, Kim MO, Zhang K, Marmer D (2009). Patients with X-linked lymphoproliferative disease due to BIRC4 mutation have normal invariant natural killer T-cell populations.. Clin Immunol.

[25] Baev DV, Peng XH, Song L, Barnhart JR, Crooks GM (2004). Distinct homeostatic requirements of CD4+ and CD4- subsets of Valpha24-invariant natural killer T cells in humans.. Blood.

[26] Berzins SP, Cochrane AD, Pellicci DG, Smyth MJ, Godfrey DI (2005). Limited correlation between human thymus and blood NKT cell content revealed by an ontogeny study of paired tissue samples.. Eur J Immunol.

[27] Kovalovsky D, Alonzo ES, Uche OU, Eidson M, Nichols KE (2010). PLZF induces the spontaneous acquisition of memory/effector functions in T cells independently of NKT cell-related signals.. J Immunol.

[28] Raberger J, Schebesta A, Sakaguchi S, Boucheron N, Blomberg KE (2008). The transcriptional regulator PLZF induces the development of CD44 high memory phenotype T cells.. Proc Natl Acad Sci U S A.

[29] Alonzo ES, Gottschalk RA, Das J, Egawa T, Hobbs RM (2010). Development of promyelocytic zinc finger and ThPOK-expressing innate gamma delta T cells is controlled by strength of TCR signaling and Id3.. J Immunol.

[30] Kreslavsky T, Savage AK, Hobbs R, Gounari F, Bronson R (2009). TCR-inducible PLZF transcription factor required for innate phenotype of a subset of gammadelta T cells with restricted TCR diversity.. Proc Natl Acad Sci U S A.

[31] Felices M, Yin CC, Kosaka Y, Kang J, Berg LJ (2009). Tec kinase Itk in gammadeltaT cells is pivotal for controlling IgE production in vivo.. Proc Natl Acad Sci U S A.

[32] Verykokakis M, Boos MD, Bendelac A, Adams EJ, Pereira P (2010). Inhibitor of DNA binding 3 limits development of murine slam-associated adaptor protein-dependent “innate” gammadelta T cells.. PLoS One.

[33] Weinreich MA, Odumade OA, Jameson SC, Hogquist KA (2010). T cells expressing the transcription factor PLZF regulate the development of memory-like CD8+ T cells.. Nat Immunol.

[34] Renukaradhya GJ, Khan MA, Vieira M, Du W, Gervay-Hague J (2008). Type I NKT cells protect (and type II NKT cells suppress) the host's innate antitumor immune response to a B-cell lymphoma.. Blood.

[35] Swann JB, Uldrich AP, van Dommelen S, Sharkey J, Murray WK (2009). Type I natural killer T cells suppress tumors caused by p53 loss in mice.. Blood.

[36] Teng MW, Sharkey J, McLaughlin NM, Exley MA, Smyth MJ (2009). CD1d-based combination therapy eradicates established tumors in mice.. J Immunol.

[37] Teng MW, Yue S, Sharkey J, Exley MA, Smyth MJ (2009). CD1d activation and blockade: a new antitumor strategy.. J Immunol.

[38] Tahir SM, Cheng O, Shaulov A, Koezuka Y, Bubley GJ (2001). Loss of IFN-gamma production by invariant NK T cells in advanced cancer.. J Immunol.

[39] van der Vliet HJ, Pinedo HM, von Blomberg BM, van den Eertwegh AJ, Scheper RJ (2002). Natural Killer T cells.. Lancet Oncol.

[40] Motohashi S, Nakayama T (2009). Invariant natural killer T cell-based immunotherapy for cancer.. Immunotherapy.

[41] Novak J, Griseri T, Beaudoin L, Lehuen A (2007). Regulation of type 1 diabetes by NKT cells.. Int Rev Immunol.

[42] Wither J, Cai YC, Lim S, McKenzie T, Roslin N (2008). Reduced proportions of natural killer T cells are present in the relatives of lupus patients and are associated with autoimmunity.. Arthritis Res Ther.

[43] Van Kaer L (2006). Role of invariant natural killer T cells in immune regulation and as potential therapeutic targets in autoimmune disease.. Expert Rev Clin Immunol.

[44] Fischer S, Kohlhase J, Bohm D, Schweiger B, Hoffmann D (2008). Biallelic loss of function of the promyelocytic leukaemia zinc finger (PLZF) gene causes severe skeletal defects and genital hypoplasia.. J Med Genet.

[45] Mitsuo A, Morimoto S, Nakiri Y, Suzuki J, Kaneko H (2006). Decreased CD161+CD8+ T cells in the peripheral blood of patients suffering from rheumatic diseases.. Rheumatology (Oxford).

[46] Kosub DA, Lehrman G, Milush JM, Zhou D, Chacko E (2008). Gamma/Delta T-cell functional responses differ after pathogenic human immunodeficiency virus and nonpathogenic simian immunodeficiency virus infections.. J Virol.

[47] Lee YJ, Jeon YK, Kang BH, Chung DH, Park CG (2010). Generation of PLZF+ CD4+ T cells via MHC class II-dependent thymocyte-thymocyte interaction is a physiological process in humans.. J Exp Med.

[48] Martin E, Treiner E, Duban L, Guerri L, Laude H (2009). Stepwise development of MAIT cells in mouse and human.. PLoS Biol.

[49] Takahashi T, Dejbakhsh-Jones S, Strober S (2006). Expression of CD161 (NKR-P1A) defines subsets of human CD4 and CD8 T cells with different functional activities.. J Immunol.

[50] Billerbeck E, Kang YH, Walker L, Lockstone H, Grafmueller S (2010). Analysis of CD161 expression on human CD8+ T cells defines a distinct functional subset with tissue-homing properties.. Proc Natl Acad Sci U S A.

[51] Turtle CJ, Swanson HM, Fujii N, Estey EH, Riddell SR (2009). A distinct subset of self-renewing human memory CD8+ T cells survives cytotoxic chemotherapy.. Immunity.

